# Molecular Mechanisms, Diagnosis and Treatments in Digestive Malignancy

**DOI:** 10.3390/ijms24076471

**Published:** 2023-03-30

**Authors:** Tatsuo Kanda, Ryota Masuzaki, Reina Sasaki-Tanaka, Hirofumi Kogure, Mitsuhiko Moriyama

**Affiliations:** Division of Gastroenterology and Hepatology, Department of Medicine, Nihon University School of Medicine, 30-1 Oyaguchi-kamicho, Itabashi-ku, Tokyo 173-8610, Japan; masuzaki.ryota@nihon-u.ac.jp (R.M.); moriyama.mitsuhiko@nihon-u.ac.jp (M.M.)

**Keywords:** transient receptor potential channel, microRNAs, telmisartan, glucagon-like peptide 1 agonist, HCC, innate immune response

In this Special Issue, “Molecular Mechanisms, Diagnosis and Treatments in Digestive Malignancy”, of the *International Journal of Molecular Sciences*, a total of 10 impactful articles have been published.

Stokłosa et al. reviewed the transient receptor potential (TRP) channel family, among ion channels, finding that it plays a role in digestive tract cancers [1]. The member of the TRP channel family have been classified into six human TRP channel subfamilies, including canonical (TRPC), melastatin (TRPM), vanilloid (TRPV), ankyrin (TRPA), polycystic (TRPP), and mucolipin (TRPML) channels. Most TRP channels play an essential role in the influx of monovalent and divalent cations, such as Na^+^, Mg^2+^, and Ca^2+^, as well as trace metal ions. Mutations in the TRP genes are not involved in most of the TRP channels’ changes in cancer. Increased or decreased levels of the expression of functional TRP proteins are involved depending on the cancer’s stage. The different expression patterns of TRP channels were observed in human oral cancer, esophageal squamous cell carcinoma (ESCC), hepatocellular carcinoma (HCC), pancreatic cancer, and gastric and colorectal cancers. It is possible that the TRP channels may be potential cancer biomarkers or therapeutic targets [1,2].

Telmisartan, an angiotensin II type 1 (AT1) receptor blocker (ARB), inhibits the proliferation of human ESCC in vitro and in vivo [3]. Telmisartan inhibits the phosphorylation of ErbB3, a member of the epidermal growth factor receptor (EGFR) family, and inhibits cell-cycle progression from synthesis (S) to the gap 2 (G2)/mitosis and cytokinesis (M) phase by decreasing the expression of cyclin A2 and cyclin-dependent kinase 2 (Cdk2) in human ESCC KYSE180 cells [3]. Telmisartan also decreases the thrombospondin-1 levels in KYSE180 cells [3]. A total of 36 and 23 MicroRNAs (miRNAs) were upregulated and downregulated, respectively, in KYSE180 cells treated with telmisartan [3]. The growth of KYSE180 tumors in nude mice was inhibited by the treatment of telmisartan [3]. Telmisartan may be effective for ESCC.

Many patients with esophageal adenocarcinoma do not benefit from chemoradiotherapy treatment due to therapy resistance [4]. miRNAs, small (19–24 nucleotides) non-coding RNA molecules, in small extracellular vesicles, comprise a stable form of circulating nucleic acids that are useful for diagnosis and as biomarkers that are predictive of treatment response [5]. Butz et al. demonstrated that the inhibition of miR-451a enhances radiosensitivity in vitro [4]. They also identified RPL13, a validated target of miR-451a, which encodes the ribosomal protein L13, as significantly upregulated after miR-451a inhibition in human esophageal adenocarcinoma OE cells. miR-451a is one of the miRNAs which was associated with the radiation unique resistance in pre-treatment serum small extracellular vesicles from patients with esophageal adenocarcinoma. let-7d-5p was the only miRNA that was associated with resistance to all three anti-neoplastic treatments (radiation, cisplatin, and 5-fluorouracil (FU)). let-7d-5p is one of the 48 miRNAs which were differently expressed after the inhibition of miR-451a [4].

Glucagon-like peptide 1 (GLP1) agonists are commonly used to treat type 2 diabetes mellitus and have shown quite promising signs of clinical efficacy in nonalcoholic steatohepatitis (NASH) [6]. Kojima et al. demonstrated that GLP1 agonists prevented the progression of HCC in a mouse model of NASH [7]. Hepatitis C virus (HCV) core protein and HCV infection, which is one of the major causes of HCC in Japan and the United States, contribute in part to the insulin resistance [8,9,10]. Thus, insulin resistance and diabetes mellitus are deeply involved in hepatocarcinogenesis [11]. Metformin treatment was associated with the reduced risk of cancers, including HCC [12,13]. Hopefully, GLP1 agonists will reduce the risk of HCC in insulin resistance or diabetes patients associated with or without HCV infection.

Enomoto et al. reviewed a possible role of the hepatoma-derived growth factor (HDGF) as a target molecule for digestive malignancies [14,15]. They previously demonstrated that the HDGF is a heparin-binding glycoprotein which has a growth-stimulating effect on hepatoma cells in vitro [16]. They also demonstrated a higher expression of the HDGF protein in cancerous tissues in comparison with the adjacent non-cancerous tissues in human HCC [16]. The HDGF is an angiogenic factor and a possible anti-apoptotic factor [15]. The HDGF is associated with the progression of esophageal cancer, HCC, cholangiocarcinoma gallbladder adenocarcinoma, pancreatic cancer, and gastric and colorectal cancers [15].

Recently, immune checkpoint inhibitors have played a central role in treating patients with unresectable HCC. The FDA-approved current standards as first-line systemic therapies for patients with unresectable HCC are either atezolizumab (anti-programmed death-ligand 1 (PD-L1)) plus bevacizumab (anti-vascular endothelial growth factor (VEGF)), or durvalumab (anti-PD-L1) plus tremelimumab (anti-cytotoxic T-lymphocyte-associated protein 4 (CTLA-4)) [17,18]. Regorafenib and lenvatinib, which are multiple kinase inhibitors used as second-line systemic therapeutic agents for patients with unresectable HCC, also had different effects on the innate immune response, including Toll-like receptor (TLR) signaling pathways, in HCC, and the modulation of the TLR signaling pathway, which will shed new light on advanced HCC patients with refractory disease [19].

It is well known that sarcopenia have an effect on the prognosis of liver cirrhosis, which most patients with HCC have. Kamimura et al. extensively reviewed the molecular mechanism and sarcopenia in liver diseases [20]. The crosstalk between liver cirrhosis and the mechanisms of muscle loss and sarcopenia, lower branched chain amino acid (BCAA) levels, hyperammonemia, and abnormal sex hormone levels, is well documented [20]. This article could help hepatologists who see patients with advanced liver disease. Both treatments for sarcopenia and liver diseases are important for the patients with advanced liver disease.

Noninvasive methods for the diagnosis of liver fibrosis are also important for the early detection of HCC [21]. Although the molecular mechanism of liver fibrosis is one of the major issues, various magnetic resonance imaging (MRI) elastography and ultrasound (US) elastography have been developed [21]. Serum biomarkers, such as the FIB-4 index, Mac-2 binding protein glycosylation isomer (M2BPGi), etc., also predict liver fibrosis easily [21,22]. Murata et al. reported that the serum novel fibrosis biomarker M2BPGi level at 48 weeks is a useful predictor with higher predictive values for HCC development in patients with chronic hepatitis B during nucleot(s)ide analogue therapy [22]. The development of new biomarkers for the early detection for HCC, the treatment response for HCC, and a prediction for the prognosis of HCC patients are in progress and will be increasingly required.

Pancreatic adenocarcinoma has a very poor prognosis among human cancers [23]. Kim et al. reported that the antioxidant transcription factor nuclear factor (erythroid-derived 2)-related factor 2 (NRF2) is a potential target for resensitizing 5-FU-resistant pancreatic ductal adenocarcinoma cells to 5-FU [24]. This strategy may confer the improvement of treatment outcomes in patients with pancreatic cancer, although the EGFR inhibitor, poly ADP ribose polymerase (PARP) inhibitor, and neurotrophic tropomyosin-receptor kinase (NTRK) inhibitors, either in combination with or without chemotherapy, are in development [25].

In conclusion, molecular mechanisms, diagnosis, and treatments for digestive malignancies and related diseases are still important issues. Further studies are required in the fight against these digestive malignancies.

## Data Availability

Data might be available from the authors of the cited papers.
